# Acetabular Revision Arthroplasty Based on 3-Dimensional Reconstruction Technology Using Jumbo Cups

**DOI:** 10.3389/fbioe.2022.799443

**Published:** 2022-04-05

**Authors:** Xianyue Shen, Hao Tian, Yang Li, Jianlin Zuo, Zhongli Gao, Jianlin Xiao

**Affiliations:** Department of Orthopedics, China-Japan Union Hospital of Jilin University, Changchun, China

**Keywords:** three-dimensional, acetabular revision, bone defect, jumbo cup, cup coverage

## Abstract

**Background:** This study was aimed at evaluating the changes in cup coverage (CC) and hip center of rotation (HCOR) in acetabular defects of various severities treated with acetabular revision using jumbo cups.

**Methods:** A total of 86 hips were included. The American Academy of Orthopedic Surgeons (AAOS) classification of these patients was as follows: 16 patients, AAOS I; 16 patients, AAOS II; and 16 patients, AAOS III. A three-dimensional (3D) implant simulation technique was used to visualize the placement of jumbo cups during revision arthroplasty. The acetabular anteversion, inclination, CC, and the HCOR were measured.

**Results:** The inclination and anteversion of simulated acetabular cups in AAOS I–III groups were consistent with the normal acetabular anatomy. Compared with the controls, in AAOS I–III groups, the HCOR was significantly increased and CC was significantly decreased. The HCOR elevation was significantly higher in AAOS III patients than in AAOS I (*p* = 0.001) and AAOS II patients (*p* < 0.001). The use of the jumbo cup technology for acetabular revision would decrease the CC in AAOS I–III patients to 86.47, 84.78, and 74.51%, respectively.

**Conclusion:** Our study demonstrated that in patients with acetabular defects, acetabular revision arthroplasty using jumbo cups will lead to decreased CC and HCOR upshift. Upon classifying these patients according to the AAOS classification, CC decreased with the severity of acetabular defects, and the elevation of the HCOR in AAOS III patients exceeded 10 mm and was significantly higher than in other patients.

## Introduction

With the widespread application of primary total hip arthroplasty (THA), there is an inevitable increase in the need for revision arthroplasty because of aseptic loosening, infection, recurrent dislocation, and periprosthetic fracture (Bozic et al., 2009; Aggarwal et al., 2019; Hoskins et al., 2020; Hoskins et al., 2021). Revision hip surgery is frequently performed for primary THA failure. Acetabular bone defects, a non-anatomical hip center, and complexities of the surgical technique typically make acetabular revision arthroplasty extremely challenging for the orthopedic surgeon (Solomon et al., 2018; Lochel et al., 2019). Some widely acknowledged acetabular bone defect evaluation systems and treatment principles, including the American Academy of Orthopedic Surgeons (AAOS) classification (D'Antonio et al., 1989) and Paprosky classification (Paprosky et al., 1994), play an important role in clinical practice. According to the anatomical morphology of the acetabular defect, the AAOS classification includes five types: segmental deficiencies, cavitary deficiencies, combined deficiencies, pelvic discontinuity and arthrodesis. The Paprosky classification was divided into three types based on the position of hip center of rotation (HCOR), the degree of tear drop damage, the degree of sciatic osteolysis, and the integrity of the Kohler line. These classification systems can evaluate the location and severity of bone defects. They have great guiding significance in clinical practice and help reconstruct acetabular bone defects and HCOR.

Presently, numerous surgical tools and strategies have been developed to resolve the dilemma caused by the acetabular defect. In this view, some of the approaches are a combination of compact grafting with cups (Green et al., 2018), jumbo cups (von Roth et al., 2015; McLaughlin and Lee, 2018), rings or cages (Innocenti et al., 2021), shells with a high HCOR (Russell et al., 2021), and cup–cage constructs (Wang et al., 2020). The use of jumbo cups is a common and effective technique to treat extensive acetabular defects. It offers the advantages of simplifying revision surgery, avoiding extensive bone grafting, and increasing the surface contact area between the cup and host bone (Lachiewicz and Watters, 2016). von Roth et al. (2015) conducted a 20-year follow-up study and reported that acetabular revision with a jumbo cup has good long-term results with regard to survival, radiographic stability, and clinical outcomes. Although several reports have been published on the results of jumbo cups use for revision arthroplasty (Dearborn and Harris, 2000; Moon et al., 2019), jumbo cups will result in native HCOR elevation as well as an unknown coverage with the host bone.

To our knowledge, few studies have reported on quantitative changes in the cup coverage (CC) and HCOR after revision surgery using jumbo cups, particularly for different grades of acetabular defects. Meanwhile, a previous study (Yang et al., 2017) has recommended the use of implant simulation technology to determine the position and effective bone mass of the acetabulum in a 3D environment. Based on the 3D implant simulation technology, we wondered whether multiple types of bone defects can be treated in the clinic using the jumbo component alone. In this study, we simulated the implantation of a jumbo cup for the treatment of acetabular bone defects to explore the generalizability and consequences of the jumbo technique.

Taken together, the primary goal of the present study was to use 3D implants with jumbo cups [Asians definition (Fan et al., 2008)] to simulate acetabular revision to elucidate 1) the changes of HCOR in AAOS I–III acetabular defects and identify whether there are any significant differences and 2) the extent of initial CC that can be achieved with a jumbo cup.

## Materials and Methods

### Patients

Between July 2015 and September 2020, 82 patients who visited our hospital for a failed acetabular cup were included. We retrospectively reviewed the preoperative computed tomography (CT) imaging data in our department. Institutional review board approval was obtained. The inclusion criteria for this study were as follows: 1) Primary THA revision involved the acetabular bone defect; 2) The revision involved unilateral hip, and the contralateral hip was normal; 3) CT imaging data were available; and 4) Jumbo cup size conformed to the definition for Asians. Of the 82 subjects, we excluded 11 subjects who had undergone THA re-revision, 10 subjects with inferior quality or no CT imaging data, and 13 subjects with revision of bilateral hips. Ultimately, we retrospectively evaluated 48 subjects (48 acetabular defects) who met the inclusion criteria. Nineteen patients (38 hips) without hip disease or deformities and with CT findings available were chosen as controls. Acetabular defects were identified by a previously described method and were classified according to the AAOS classification system (D'Antonio et al., 1989). Accordingly, 16 acetabular defects were graded as AAOS I, 16 as AAOS II, and 16 as AAOS III. Demographic data for the subjects are shown in Table 1.

**TABLE 1:** Demographic characteristics.

Subgroup	Numbers of Hips	Males/Females (No. of Hips)	Age (years)
Normal	38	24/14	38.3 ± 15.1 (31.0–45.6)
Subjects	48	29/19	59.8 ± 11.2 (56.5–63.0)
AAOS I	16	11/5	58.3 ± 11.6 (52.1–64.4)
AAOS II	16	8/8	63.3 ± 11.1 (57.4–69.2)
AAOS III	16	10/6	57.7 ± 10.5 (52.1–63.3)

Data are presented as mean and standard deviation, with the 95% confidence interval in parentheses.

### CT Protocol and Pelvic 3D Reconstruction

Pelvic CT scans including the entire pelvis and proximal femur were obtained using a Toshiba Aquilion CT scanner (120 kVp, 320 mA, 512 × 512 matrix, and 0.5-mm slice thickness). All CT slices were saved in the Digital Imaging and Communications in Medicine format and imported into Mimics 19.0 software (Materialise NV, Leuven, Belgium) for 3D reconstruction. A metal artifact-minimizing protocol was used where possible by adjusting the gray level of the image, manually identifying the metal artifact, and restoring the existing structure. The acetabular prosthesis was removed on the premise of fully retaining the host bone.

### Determination Pelvic Position and Height of HCOR

**FIGURE 1:**
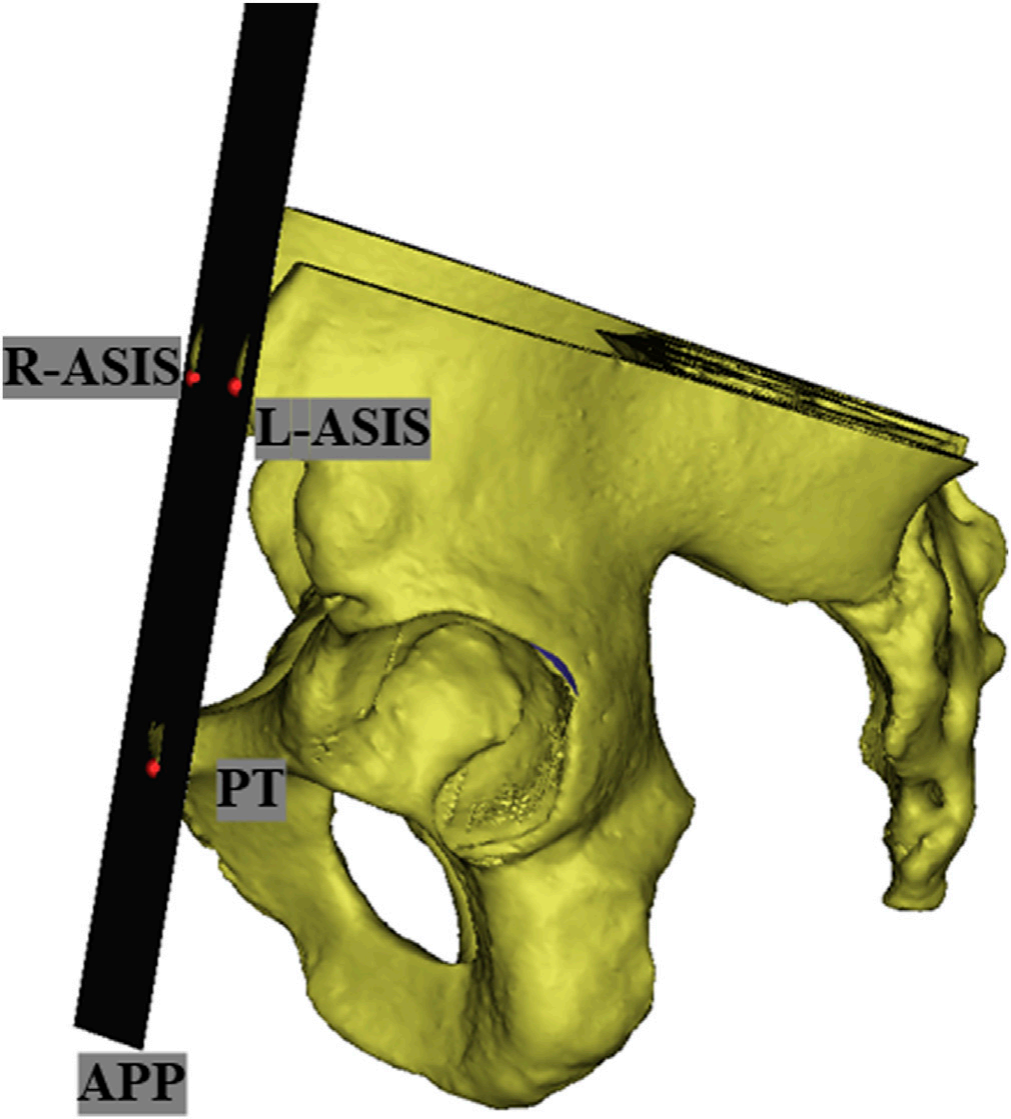
In the three-dimensional simulation, according to the left anterior superior iliac spines (L-ASIS), right anterior superior iliac spines (R-ASIS), and pubic tubercles to determine the anterior pelvic plane (APP).

Before the simulated implantation, the pelvic position was standardized with reference to the anterior pelvic plane defined by the bilateral anterior superior iliac spines and the pubic tubercles (Fujii et al., 2012; Yang et al., 2017) (Figure 1). The center of the jumbo cup is regarded as the HCOR, and rotation center of the contralateral hip was determined by Mose technique (Mose, 1980).

### Simulating Implantation Technique

According to the revision acetabular cups, a set of virtual acetabular cups was created using 3-matic 11.0 software (Materialise). The acetabular cups had a 4-mm shell thickness, and the diameter ranged from 50 to 70 mm in 2-mm intervals; then, these 3D models were imported into Mimics software in the stereolithography (STL) format.

In the 3D simulation, the inferior edge of the virtual cup should be flush with the obturator level to the extent possible to mimic the installation of the cup in a clinic. The simulated acetabular cup was marginally adjusted to reconstruct native anteversion and achieve cup inclination of 40° ± 10° within the allowable range of the real surgery, so as to maximize the preservation of the natural bone of the acetabulum. The jumbo cup size was chosen to best accommodate the anteroposterior diameter of the acetabulum with bone defect. This diameter could be adjusted to achieve maximum bone contact. The central point of the cup was considered as the HCOR.

### Evaluations and Measurements

**FIGURE 2:**
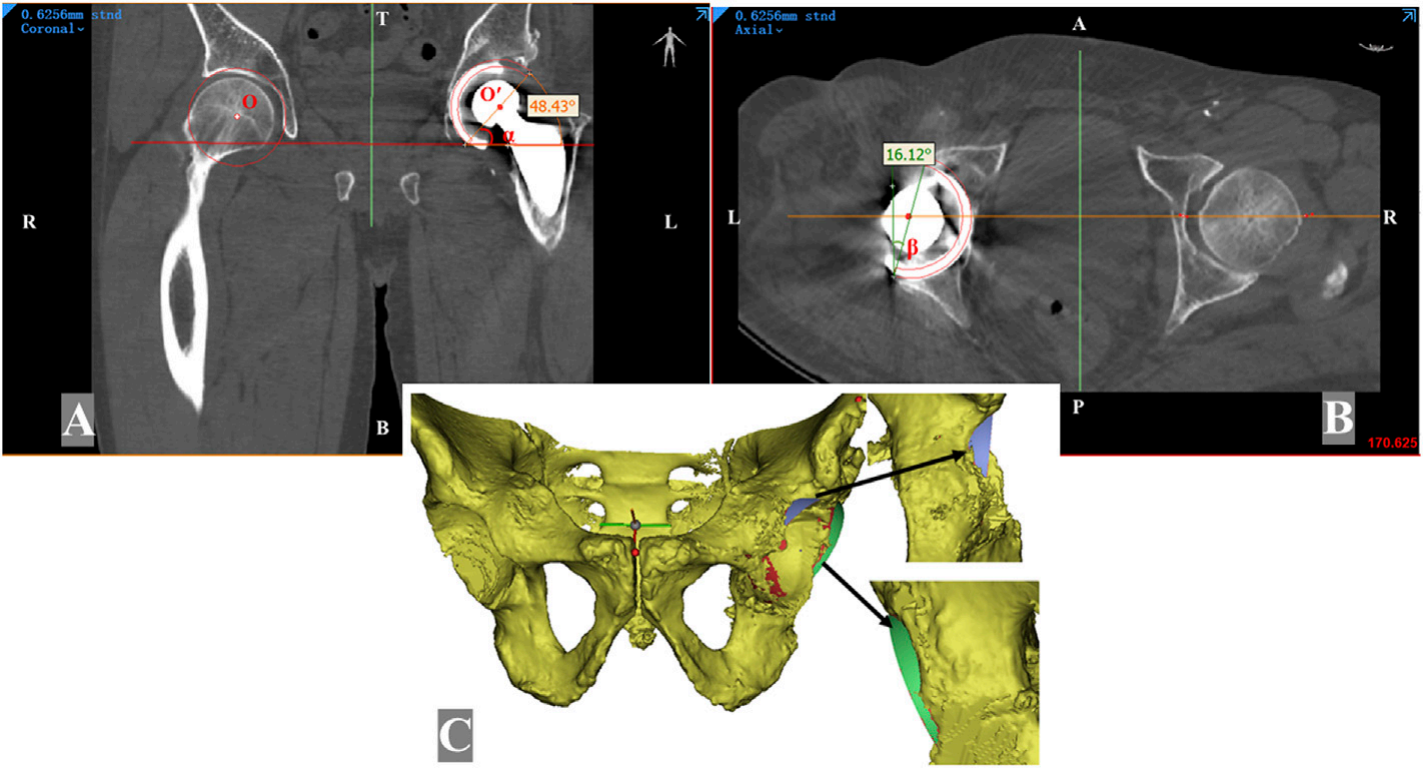
Measurement of the simulated implanted acetabular cup on the Mimics software. The reoriented planes **(A, B)** are resliced from the anterior pelvic plane. The hip center of rotation on the implanted acetabular cup side is marked as point O; the contralateral normal center of rotation is determined by Mose technique and marked as point O′. The vertical distance between point O and point O′ is the hip rotation center elevation. The inclination angle (*α*) was measured in the coronal plane **(A)**, and the anteversion angle (*β*) was measured in the axial plane **(B)**. **(C)** Segmentation was performed according to the border between the covered part (red region) and the uncovered part (blue region and green region) of the acetabular cup.

The contact surface area between the acetabular cup and native bone was determined as the effective bone mass, and sufficient bone mass was essential for the initial stability of the implanted cup. On the basis of the implantation simulation, uncovered area (Su) of the acetabular cup was defined as the area of the surface uncovered by the native bone. The total acetabular cup surface area (St) represented the area available for surface contact. CC was calculated as the ratio of (St—Su) to St (Figure 2).

The distribution map of the uncovered area of the jumbo cup was created to visualize the localization of the missing bone contact. After implantation simulation, the uncovered area was delineated on the acetabular component and exported in the STL format. Then, these files were imported into Magics 22.03 software (Materialise), the uncovered areas of all sizes of cups were unified into the same side by mirror image processing and standardized to 60-mm acetabular component. All uncovered areas were overlapped on the 60-mm acetabular model to create a compilation of the uncovered areas and generate a visual heat map. To easily indicate the specific location of the loss of bone contact, a clock diagram was made according to the opening orientation of the acetabular cup.

The height difference of the HCOR was defined as the height difference between the jumbo cup and the contralateral hip center. The calculation was performed as follows. First, we identified the inferior edge of the teardrops on both sides on the coronal plane to obtain the inter-teardrop line, then the distance between both hip centers and the inter-teardrop line was measured. If unilateral teardrop osteolysis was noted, a mirror image of the contralateral teardrop was used to replace it. Furthermore, bilateral acetabular anteversion and inclination were measured in axial and coronal images, individually (Figure 2).

### Statistical Analysis

Statistical analysis was performed using SPSS 22.0 (IBM, Chicago, IL, United States). The significance level was set at *p* < 0.05, which was tested using the independent-samples Student *t* test. All measurements were performed by two experienced surgeons simultaneously. We used G*Power software version 3.1.9.7 (Medistat, Kiel, Germany)) to calculate the sample size of the study. Based on a confidence level of 95% (*α* = 0.05) and a power (1–β) of 80%, effect size = 0.43 which was calculated from our pilot tests, number of groups = 4, a total sample size of 64 samples was required. Therefore, at least 16 samples were required for each subgroup. The measurements were then repeated by one of the two surgeons with a minimum interval of 4 weeks since the previous measurement. This helped evaluate inter- and intraobserver reliability. A one-way random effects model of the intraclass correlation coefficient (ICC) was used to quantify interobserver and intraobserver reliability of the measurements to assess the reproducibility. A reliability coefficient >0.75 was considered to indicate good reliability.

## Results

The use of 3D implant simulation technology is promising for the evaluation and preoperative planning of acetabular revision arthroplasty with acetabular defects. The size and position parameters of the acetabular cups are shown in Table 2. The size of the implanted acetabular cups was significantly larger in the AAOS I–III groups than in the control group (*p* < 0.001). We found the inclination and anteversion of the simulated acetabular cups in the AAOS I–III groups to be mostly consistent with normal acetabular anatomy, except for the inclination in the AAOS–III group, which was significantly greater than that in the control group (44.40° ± 2.53° vs. 47.23° ± 2.55°; *p* = 0.001). The remaining position parameters did not statistically significantly differ among the subgroups.

**TABLE 2:** The size and position parameters of implanted acetabular cups in 3-Dimensional environment.

Subgroup	Number of Hips	Acetabular Cup		
		Size (mm)	Acetabular Anteversion (°)	Acetabular Inclination (°)
Normal	38	50.95 ± 3.08 (49.46–52.43)	16.83 ± 2.85 (15.89–17.77)	44.40 ± 2.53 (43.57–45.23)
Subjects	48	61.58 ± 3.98 (60.43–62.74)	16.85 ± 3.04 (15.97–17.73)	46.17 ± 2.52 (45.37–46.97)
AAOS I	16	61.25 ± 3.09 (59.60–62.90)**	17.16 ± 2.49 (15.84–18.49)	45.52 ± 3.13 (43.85–47.19)
AAOS II	16	62.13 ± 3.76 (60.12–64.13)**	16.59 ± 3.43 (14.76–18.42)	46.74 ± 2.38 (44.48–47.01)
AAOS III	16	61.37 ± 5.04 (58.69–64.06)**	16.79 ± 3.27 (15.05–18.53)	47.23 ± 2.55 (45.88–48.59)**

Data are presented as mean and standard deviation, with the 95% confidence interval in parentheses. *Represents compared with the control group with *p* < 0.05, **Represents *p* < 0.01.

**FIGURE 3:**
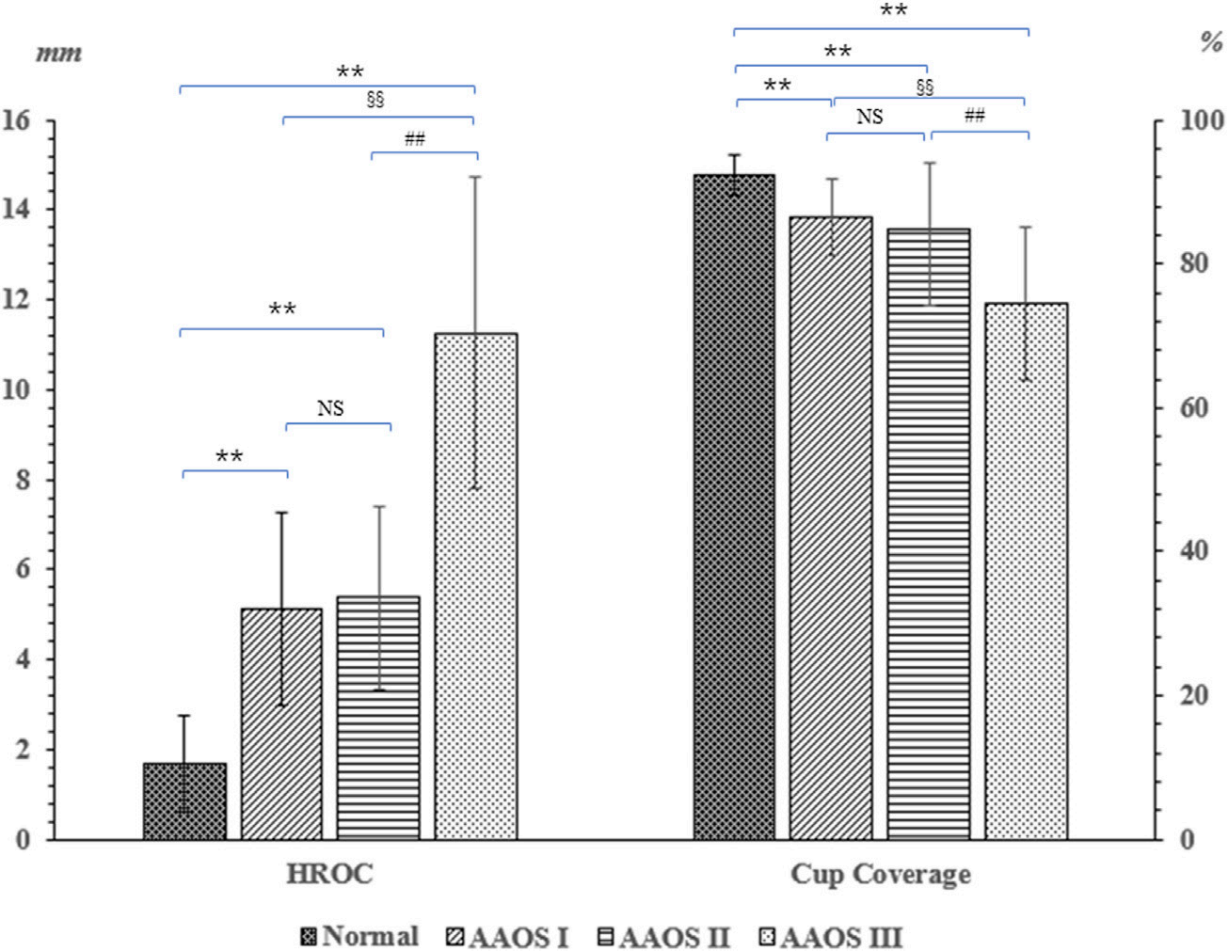
Comparison of the implanted acetabular cup hip rotation of center elevation and cup coverage in the control and AAOS I–III groups. Represents compared with the control group with *p* < 0.05, ** Represents *p* < 0.01. ^#^ *p* < 0.05 represents the statistical difference between the AAOS II and AAOS III; ^##^ Represents *p* < 0.01. ^§^ *p* < 0.05 Represents the statistical difference between the AAOS I and AAOS III; ^§§^ Represents *p* < 0.01.

Our 3D implant simulation results show that the jumbo cup technology can lead to HCOR elevation and provide satisfactory initial acetabular CC (Table 3). Compared with the control group, the HCOR of AAOS I–III groups was significantly increased (1.68 ± 1.07 mm vs. 7.24 ± 3.85 mm; *p* < 0.001). Our results showed that the heights of the HCOR in AAOS I–III were upshifted by 5.11, 5.37, and 11.25 mm, respectively. The independent-samples Student *t* test of these data showed that the HCOR elevation was significantly higher in the AAOS III group than in AAOS I (*p* = 0.001) and AAOS II groups (*p* < 0.001; Figure 3). Furthermore, our results showed that the use of the jumbo cup technology for acetabular revision gradually reduced the CC in AAOS I–III groups to 86.47, 84.78, and 74.51%, respectively. In addition, we found a statistically significant difference between AAOS I and AAOS III groups in this regard (*p* = 0.002).

**TABLE 3:** Hip center and cup coverage measurements of acetabular cup.

Subgroup	Number of Hips	Hip Center Elevation (mm)	Cup Coverage (%)
Normal	38	1.68 ± 1.07 (1.17–2.20)	92.35 ± 2.77 (91.44–93.26)
Subjects	48	7.24 ± 3.85 (6.13–8.36) **	81.92 ± 10.06 (79.00–84.84) **
AAOS I	16	5.11 ± 2.14 (3.97–6.25) **	86.47 ± 5.34 (83.62–89.32) **
AAOS II	16	5.37 ± 2.05 (4.27–6.46) **^, ##^	84.78 ± 9.26 (79.84–89.72) **^,^^##^
AAOS III	16	11.25 ± 3.46 (9.41–13.10) **^, §§^	74.51 ± 10.66 (68.83–80.19) **^,^^§§^

Data are presented as mean and standard deviation, with the 95% confidence interval in parentheses. *Represents compared with the control group with *p* < 0.05; ** Represents *p* < 0.01. ^&^ Represents the statistical difference between AAOS Ⅰ and AAOS Ⅱ with *p* < 0.05; ^&&^ Represents *p* < 0.01. #*p* < 0.05 Represents the statistical difference between AAOS II and AAOS III; ^##^ Represents *p* < 0.01. ^§^ *p* < 0.05 Represents the statistical difference between AAOS I and AAOS III; ^§§^ Represents *p* < 0.01.

**FIGURE 4:**
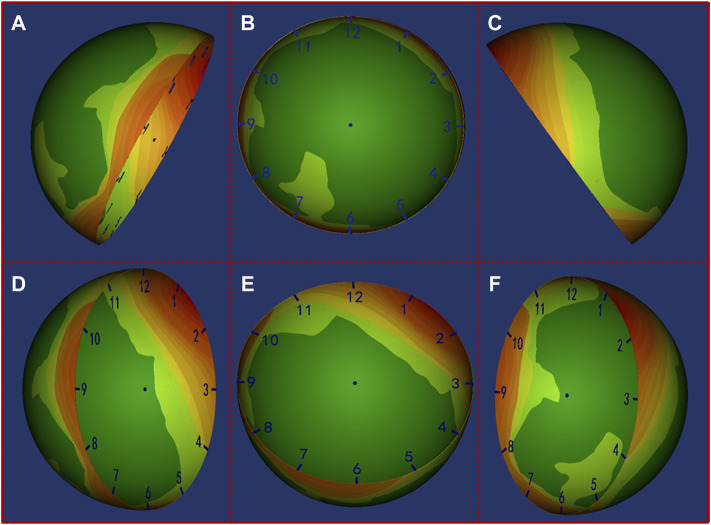
Distribution map of the uncovered area in AAOS I group. Green to red indicates that the uncovered frequency ranges from low to high. **(A)** Front view of the pelvis, **(B)** opening orientation view of the cup, **(C)** Back view of the pelvis, **(D)** Lateral view of the cup from front to back, **(E)** Lateral view of the cup from bottom to top, **(F)** Lateral view of the cup from back to front.

**FIGURE 5:**
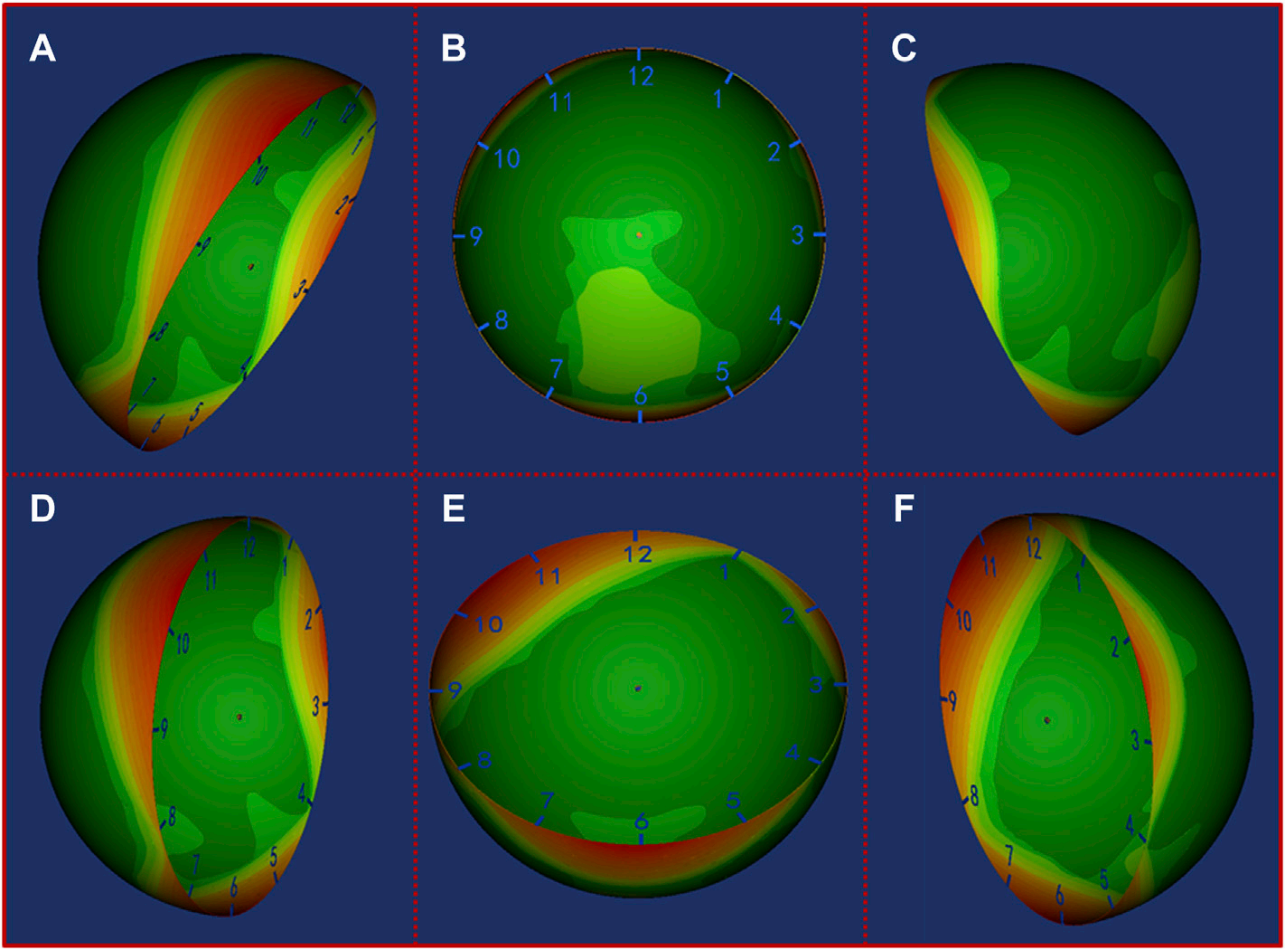
Distribution map of the uncovered area in AAOS II group. Green to red indicates that the uncovered frequency ranges from low to high. **(A)** Front view of the pelvis, **(B)** opening orientation view of the cup, **(C)** Back view of the pelvis, **(D)** Lateral view of the cup from front to back, **(E)** Lateral view of the cup from bottom to top, **(F)** Lateral view of the cup from back to front.

**FIGURE 6:**
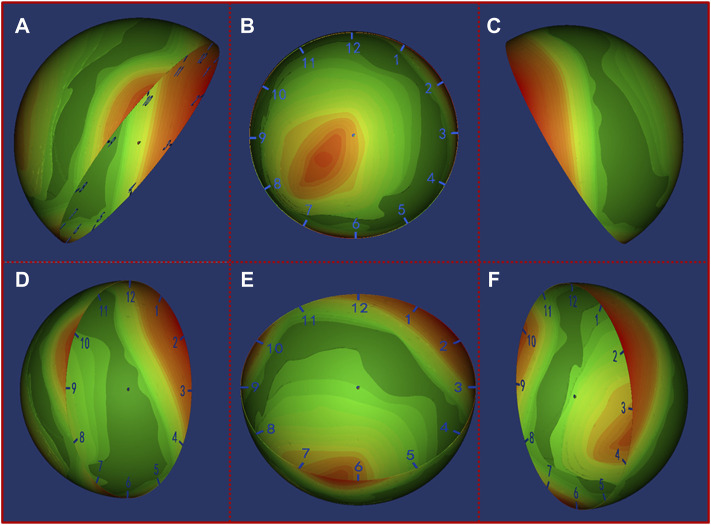
Distribution map of the uncovered area in AAOS III group. Green to red indicates that the uncovered frequency ranges from low to high. **(A)** Front view of the pelvis, **(B)** opening orientation view of the cup, **(C)** Back view of the pelvis, **(D)** Lateral view of the cup from front to back, **(E)** Lateral view of the cup from bottom to top, **(F)** Lateral view of the cup from back to front.

Based on the heat map results, the localization of the missing bone contact could be clearly visualized. Our results showed that for AAOS I group undergoing acetabular revision with a jumbo cup, the location of missing bone contact was mainly concentrated in the posterior wall (1 o’clock to 3 o’clock) and anterior wall (8 o’clock to 10 o’clock) of the acetabulum (Figure 4). For AAOS II group, the results showed that the location of the bone defect was mainly concentrated in the anterosuperior wall (8 o’clock to 12 o’clock) (Figure 5). Conversely, in AAOS III group, it was mainly concentrated in the posterosuperior wall (12 o’clock to 4 o’clock) and the bottom of the acetabulum (Figure 6).

As shown in Table 4, ICC results for intra- and interobserver reliabilities were in the excellent range.

**TABLE 4:** Intraobserver and Interobserver reliability for all acetabular cup measurements.

Measurements	Intraobserver Intraclass Correlation Coefficient	Interobserver Intraclass Correlation Coefficient
Anteversion	0.947 (0.870–0.979)	0.875 (0.710–0.950)
Inclination	0.947 (0.869–0.979)	0.896 (0.755–0.958)
Hip center elevation	0.909 (0.784–0.964)	0.880 (0.720–0.952)
Cup coverage	0.982 (0.956–0.933)	0.928 (0.826–0.971)

Data are presented as intraclass correlation coefficients, with 95% confidence interval in parentheses.

## Discussion

Some studies (Sariali et al., 2016; Yang et al., 2017; Liu et al., 2018) have proven that for complex anatomical deformities of the hip, such as developmental dysplasia of the hip (DDH) and acetabular bone defects, CT imaging data-based preoperative 3D planning can help with 1) comprehensively and accurately understanding the extent of the lesion and accordingly determining the implant location of the prosthesis, 2) pre-evaluation of the potential peri- and postoperative risks, and 3) improving the surgical effect. Acetabular revision arthroplasty for patients with moderate-to-extensive bone defects is often difficult for orthopedic surgeons, and using a jumbo acetabular cup is a relatively straightforward procedure for revision (von Roth et al., 2015). The use of jumbo cups in acetabular revisions can provide a larger circumference for ring fixation and a larger contact surface area to enhance bone ingrowth with the host bone. However, it would result in compromised CC and HCOR elevation. The present study revealed two principal findings. First, acetabular revision arthroplasty with jumbo cups for the treatment of AAOS I–III acetabular bone defects can lead to significant HCOR elevation, particularly for AAOS III, wherein the elevation is > 10 mm (11.25 ± 3.46 mm). Second, the contact area between the jumbo cup and the host bone can be accurately quantified by 3D simulation implantation technology. We found that the average CC obtained with the jumbo cup technology in patients with AAOS I–III acetabular bone defects was not less than 70%, which may provide good initial cup stability and prevent early loosening.

Ideally, the acetabular prosthesis should be placed in the living host bone as close as possible to the anatomical HCOR. However, this may be difficult or even impossible in patients with severe acetabular bone defects. In this study, we found that acetabular revision arthroplasty with the jumbo cup technology upshifted the HCOR and provided better initial contact between the jumbo cup and the host bone. This is in line with the application effect of high hip center technology in the treatment of complex anatomical deformities of the hip (Liu et al., 2018; Montalti et al., 2018).

Several previous studies have reported that acetabular revision with the jumbo cup technology can elevate the HCOR (Nwankwo and Ries, 2014; Zhou et al., 2018; Zhang et al., 2019). For instance, Nwankwo and Ries (2014) retrospectively analyzed the X-ray radiographic images of 98 patients having undergone cementless jumbo cup acetabular revisions and found that acetabular revision arthroplasty with jumbo cups upshifted HCOR by approximately 11 mm. Nevertheless, our results reported that the average HCOR elevation in patients with acetabular bone defects who underwent acetabular revision arthroplasty was 7.24 mm, indicating a lesser elevation than that reported by Nwankwo et al. This difference could be attributed to them not having taken into account the severity of the acetabular bone defect. To our surprise, after stratifying the acetabular bone defect using the AAOS classification, we found that only AAOS III patients showed an HCOR upshift of approximately 11.25 mm, which is in agreement with Nwankwo et al.’s findings. In a retrospective study, Zhang et al. (2019) included 61 patients who underwent acetabular revision using jumbo cups and measured the height of HCOR in 42 patients with a normal contralateral hip. The mean HCOR elevation in the revision side was found to be approximately 8.2 mm higher than that on the normal side, which is in line with our findings.

Bringing the HCOR to a more anatomical position during revision surgery may improve the biomechanics of the hip (Patel et al., 2003; Galia et al., 2017). Acetabular revision with the jumbo cup is evidently not conducive to HCOR anatomical reconstruction and is regarded as a surgical procedure that affects hip biomechanics, which has caused surgeons’ concerns. Johnston et al. (1979) were the first to design a mathematical model of the hip to evaluate the mechanical changes, and they showed that the hip load was the greatest when the HCOR was placed laterally, superiorly, and posteriorly. However, they did not focus on the results of simple upshift of HCOR. Based on a 3D computer model, Delp et al. (1996) found that superolateral placement of the HCOR resulted in an average reduction 28% in the abductor muscle moment arm. This could lead to muscle imbalance and prosthetic joint dislocations (Renkawitz et al., 2016; Hu et al., 2021). Further, Bicanic et al. (2009) quantified the relationship between the displacement of HCOR and the joint response force in the experiment and found that upshifting the HCOR by 1 mm will increase the joint response force by 0.1%. This also reminds us that upshifting the HCOR will increase the load pressure, thus increasing the wear of the prosthesis due to increased friction at the interface. In addition, the HCOR elevation will lead to differences in the leg length and postoperative kinematics (Karaismailoglu et al., 2019; Hu et al., 2022). In a retrospective study, Dou et al. (2013) found that acetabular revision increased HCOR elevation, thus resulting in leg length differences. Hu et al. (2022) found that laterally or superiorly placed HCOR would increase abnormal extension and internal rotation, resulting in impaired gait patterns. These aspects need attention, especially for largely HCOR elevation in acetabular revision.

However, the current study confirmed that the use of the jumbo cup still leads to a HCOR upshift, particularly in AAOS III patients, which reminds surgeons that the use of the jumbo cup technology in more severe acetabular bone defects requires in-depth consideration as it may change hip biomechanics, increase the risk of component loosening, and increase the need of re-revision surgery (Delp et al., 1996; Peng et al., 2021). Of course, these negative effects can be averted through technological advancements in the development of prostheses, such as improving the friction interface (Hu et al., 2015) and increasing the length of the femoral head (Woelfle et al., 2014). It is encouraging that some authors have reported good clinical results using jumbo cups with improved manufacturing processes (Fan et al., 2008; Warschawski et al., 2021).

Increasing the contact area between the acetabular cup and the host bone can ensure good initial stability, which is a crucial success factor of hip arthroplasty revision. CC is usually used to evaluate this contact area and in DDH hip replacement (Yang et al., 2017; Liu et al., 2018; Mou et al., 2020; Takasago et al., 2021). Generally, insufficient CC will increase the stress at the bone cup interface, thus increasing the probability of mechanical failure (Apostu et al., 2018; Zuo et al., 2021). Several clinical studies have shown that a CC value of ≥70% is acceptable and can ensure initial stability and prevent earlier loosening (Xu et al., 2012; Li et al., 2013). In the CT analysis study, Liu et al. (2018) found that the high hip center technology can effectively increase CC in DDH Crowe Type III patients. The HCOR is moved up by 25 mm and can provide a CC value of nearly 85%. To our knowledge, no study has quantitatively evaluated acetabular CC in hip revision surgery using a jumbo cup. We found in our 3D simulation study that with bone defect aggravation, the CC of the acetabulum gradually decreased; however, HCOR elevation remained significantly higher in AAOS III patients than in AAOS I and AAOS II patients with acetabular bone defects. It is reassuring that CC reached >70% even in AAOS III patients. This also explains the great prospects brought about by the use of the jumbo cup technology in increasing bone contact of the host bone with bone trabecular metal, thus potentially improving the ingrowth and ongrowth ability of host bone (Shen et al., 2022).

This study has some limitations. First, relatively few patients were included, resulting in a fewer subitems distribution of AAOS I and AAOS II (segmental defect and cavity defect), prevented further detailed subitems analysis in acetabular revision using jumbo cups. But the results still showed significant differences in the overall classification. Second, AAOS IV patients with pelvic discontinuity were not included, mainly due to the short study time span and the rarity of AAOS IV patients. For pelvic discontinuity, however, the clinical treatment principle is to use the acetabular cup to obtain initial stabilization with bone press-fit contact between the anterosuperior and posteroinferior acetabulum to reconstruct the discontinuity of the acetabulum. It is inappropriate to use the CC evaluation, which is different from AAOS I–III acetabular defect. Therefore, this will not affect our conclusion. Third, this study is only a performed 3D simulation study with a singular focus on imaging parameters, which may not fully represent the surgical situation. Nevertheless, these imaging indicators are of great significance, which is conducive to making preoperative plans and avoiding revision failure-related complications in complex situations. In addition, the selected acetabular cup size is determined by the remaining bone stock. In some cases, the anteversion may be compromised to obtain better CC even if the average anteversion angle of the implanted acetabular cup was normal.

## Conclusion

In summary, although the use of jumbo cups for acetabular revision arthroplasty in patients with AAOS I–III offers reduced initial CC with increasing severity of the acetabular bone defect, the CC consistently remained >70%. In addition, it is associated with significant elevation of >10 mm of the HCOR in AAOS III patients. This information may remind the surgeons additional procedures are needed to compensate for the elevation of the hip center using jumbo cups.

## Data Availability

The raw data supporting the conclusion of this article will be made available by the authors, further inquiries should be directed to the corresponding author.
